# Supplementary material for: Fidelity of a Motivational Interviewing Intervention for Improving Return to Work for People with Musculoskeletal Disorders
Source: Int J Environ Res Public Health. 2021 Sep 30;18(19):10324. doi: 10.3390/ijerph181910324 (PMC8507704; doi:10.3390/ijerph181910324)
Supplement: Supplementary file 1 [file ijerph-18-10324-s001.zip › ijerph-1382470-supplementary.pdf]

Table S1

Checklist for adherence to the MI manual based on the main themes

MI session 1		Adherence (0-4)*
1	Introduction of session 1 from the interviewer – the frame of the conversation	
2	Agenda mapping: the sick listed person (SLP) chooses theme(s) from a sheet for agenda mapping (e.g family, mental health, economy, etc.)	
3	The interviewer adapt the intervention to the sick-listed`s stage of motivation for RTW	
4a.	Rating scale about the SLP`s importance of being able to work and follow-up questions	
4b.	Rating scale about the SLP `s confidence in being able to work and follow-up questions	
5	The interviewer provides a summary of session 1 to the SLP	
Total score (mean, SD)		

*0 = not at all 1= to a small degree 2= to some degree 3=to a large degree 4= to a very large degree

MI session 2		Adherence (0-4)*
6	Introduction and summary of session 1 (previous conversation)	
7	Introduction to session 2 from the interviewer	
8	Assess the SLP`s current work situation	
9	Assess the SLP`s previous and / or possible work attempts	
10	Information on possible contributions from the Norwegian Labour and Welfare Administration (NAV)	
11	Rating scale about the SLP`s confidence in being able to work and follow-up questions	
12	Explore the SLP`s goals and values in life	
13	Scaling of readiness for RTW and follow- up questions	
14	Summary of both session 1 and 2 with key questions	
15	Intervention in the last part of the conversation adapted to the stage of motivation	
16	Thanking the participant for the conversation	
Total score (mean, SD)		

*0 = not at all 1= to a small degree 2= to some degree 3=to a large degree 4= to a very large degree
